# Learning drug synergy through environment-conditioned feature modulation

**DOI:** 10.1093/bioinformatics/btag256

**Published:** 2026-05-05

**Authors:** Shuting Jin, Anqi Huang, Yajie Meng, Zhonghang Zhu, Yinghui Jiang, Junlin Xu, Xiangxiang Zeng

**Affiliations:** School of Computer Science and Technology, Wuhan University of Science and Technology, Wuhan, Hubei 430065, China; Hubei Province Key Laboratory of Intelligent Information Processing and Real-time Industrial System, Wuhan, Hubei 430065, China; School of Computer Science and Technology, Wuhan University of Science and Technology, Wuhan, Hubei 430065, China; School of Computer Science and Artificial Intelligence, Wuhan Textile University, Wuhan, Hubei 430200, China; School of Electronic Information, Wuhan University of Science and Technology, Wuhan, Hubei 430081, China; School of Informatics, Xiamen University, Xiamen, Fujian 361102, China; School of Computer Science and Technology, Wuhan University of Science and Technology, Wuhan, Hubei 430065, China; Hubei Province Key Laboratory of Intelligent Information Processing and Real-time Industrial System, Wuhan, Hubei 430065, China; College of Computer Science and Electronic Engineering, Hunan University, Changsha, Hunan 410082, China; The Ministry of Education Key Laboratory of Fusion Computing of Supercomputing and Artificial Intelligence, Hunan University, Changsha, Hunan 410082, China

## Abstract

**Motivation:**

Drug combinations are crucial for overcoming resistance in cancer therapy. Although deep learning has achieved strong performance in synergy prediction, existing models often treat cell-specific features and paired drugs as a static background and fail to capture how the specific cell-drug environment dynamically modulates drug representations, thereby hindering the modeling of environment-specific synergistic effects.

**Results:**

We propose Env-Syn, a framework for modeling drug-drug-cell interactions through Environment-Conditioned Feature Modulation, which incorporates a Residual Feature-wise Linear Modulation (R-FiLM) module to perform precise affine transformations on drug representations conditioned on paired drugs and cellular environments. Benchmark evaluations show that Env-Syn consistently outperforms state-of-the-art methods. Notably, the model exhibits exceptional generalization performance in rigorous inductive scenarios. It maintains high predictive accuracy for unseen drugs with AUROC and AUPRC exceeding 0.81 in the Leave-drug-out setting and further demonstrates strong cross-dataset reliability by surpassing a recall of 0.7 on independent test set. Furthermore, among 15 novel predicted drug combinations, 8 are directly supported by literature evidence. These results demonstrate that Env-Syn is an effective computational tool for drug synergy discovery.

**Availability and implementation:**

The source code is available at https://github.com/AnQi-87/Env-Syn.

## Graphical Abstract

**Graphical Abstract:**
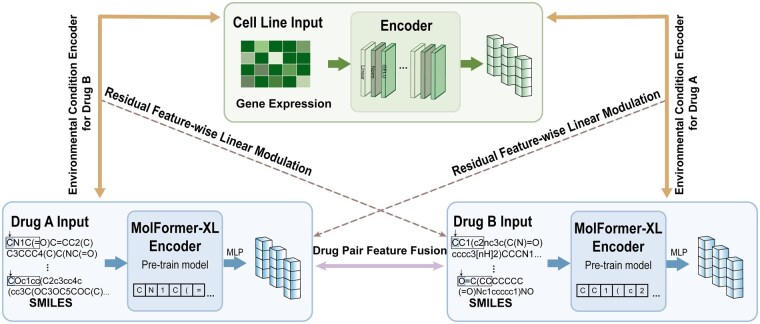

## 1 Introduction

Complex diseases such as cancer exhibit high heterogeneity and evolving molecular mechanisms, posing severe challenges for monotherapy in sustaining long-term efficacy and preventing drug resistance (Ge *et al.* 2025). Drug combinations offer significant potential to enhance therapeutic efficacy and reduce toxicity by synergistically modulating multiple targets and pathways (Jang and Chang 2025, Raut and Sharma 2025). With the advancement of high-throughput screening technologies, large-scale datasets have emerged to support the development of computational prediction models (O’Neil *et al.* 2016, Zagidullin *et al.* 2019). However, faced with the combinatorial explosion of potential drug candidates, there is an urgent need in precision medicine to develop more efficient and accurate computational models to predict drug synergy and identify optimal drug combinations (Martin 2021).

Deep learning (DL), with its advantages in high-dimensional nonlinear representation, has achieved significant strides in drug synergy prediction (Baptista *et al.* 2023). Existing models, such as DeepSynergy (Preuer *et al.* 2018) and DeepDDS (Wang *et al.* 2022), integrate drug molecular structures with cell line multi-omics features, utilizing neural networks to capture complex nonlinear interactions between drugs and biological systems. Furthermore, the integration of protein–protein interaction (PPI) networks and knowledge graphs has further enhanced the capacity to model implicit associations within multi-source heterogeneous data (Rafiei *et al.* 2023, 2024).

Despite these advancements, existing drug synergy prediction models still face core challenges. First, most models fail to adequately account for the conditional dependence of a drug under the joint influence of another drug and the specific cellular environment. Second, cell line features and paired drugs are typically treated as static global background information, failing to achieve conditional feature modulation of drug representations in specific environments. This limitation constrains the generalization and accuracy of models when confronting complex biological scenarios.

**Figure 1:**
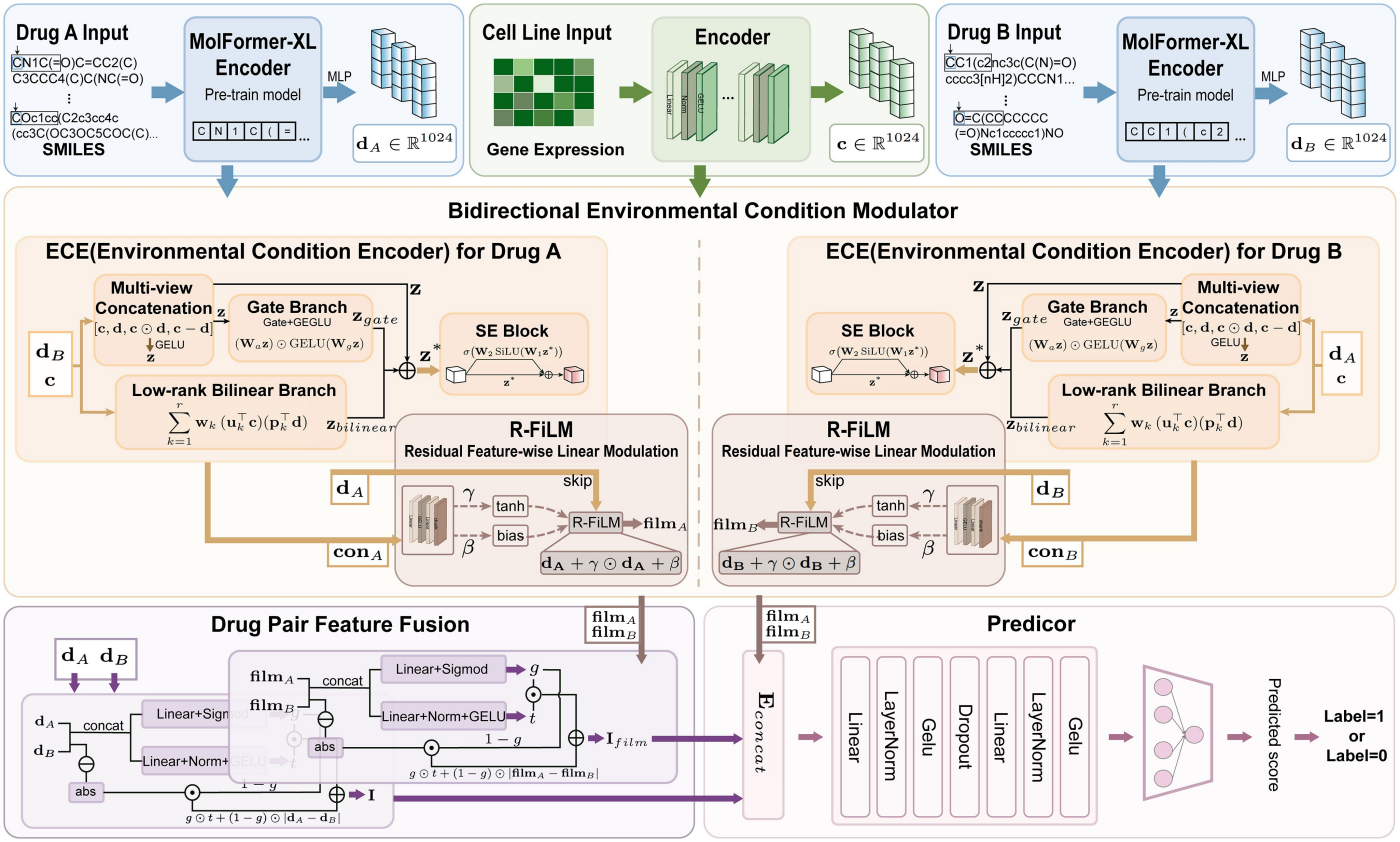
Overview of the Env-Syn framework. **Inputs**, the model first extracts feature representations from Drug A, Drug B, and Cell Line gene expressions using the MolFormer-XL Encoder and a dedicated Encoder. **Bidirectional Environmental Condition Modulator**, integrates the environment of the cell line and the partner drug into each drug’s representation using Environmental Condition Encoders (ECE) and R-FiLM modules. **Drug Pair Feature Fusion**, combines the original and modulated features, which are then passed through the Predictor. **Predictor**, outputs the final drug synergy score and classification label.

To address these challenges, we propose Env-Syn, an environment-aware framework for drug synergy prediction, as illustrated in Fig. 1. The core of Env-Syn lies in explicitly modeling the conditional representations within ternary interactions. We introduce the Residual Feature-wise Linear Modulation (R-FiLM) module, which enables drug features to undergo precise affine transformations guided by modulation parameters derived from the joint environmental context of the paired drug and the specific cell line (Perez *et al.* 2018). This design preserves the intrinsic chemical information of the drugs while empowering the model to flexibly adjust feature scales and biases according to specific environmental conditions. Additionally, a gated adaptive fusion mechanism is incorporated to enhance the representation of complex interaction patterns. Experimental results demonstrate that Env-Syn significantly outperforms state-of-the-art models on benchmark datasets. Notably, in inductive settings, both Leave-one-out validation and independent validation further highlight its strong generalization capability. Moreover, among 15 predicted novel drug combinations, 14 are supported by existing literature evidence, including 8 directly validated combinations and 6 mechanistically consistent combinations, demonstrating its potential to advance drug discovery and precision medicine.

## 2 Materials and methods

### 2.1 Data preprocessing and feature extraction

#### 2.1.1 Molecular feature extraction

We employ the pre-trained model MolFormer-XL (Ross *et al.* 2022) to extract molecular representations from SMILES sequences. Unlike 2D graph structures that primarily focus on local connectivity, the Transformer-based MolFormer-XL is pre-trained on a massive scale of 1.1 billion molecules. This allows the model to capture high-level chemical semantics and long-range dependencies within the SMILES sequence that are often critical for understanding global molecular properties. Furthermore, this sequence-based approach avoids the computational overhead associated with explicit graph construction while maintaining robust expressive power.

First, all input SMILES strings are canonicalized. Following the standard preprocessing used in MolFormer pretraining, we use RDKit (Landrum *et al.* 2006) to convert SMILES into canonical form to ensure that different SMILES representations of the same molecule map to the same string. For example, different representations of ethanol (e.g., “CCO,” “OCC”) are all standardized to “CCO.”

For each molecule represented by a canonical SMILES string *S*, we tokenize the sequence using the MolFormer tokenizer, producing a token sequence t. The token sequence is then fed into the pre-trained MolFormer encoder to obtain continuous vector representations:


(1) $$\begin{array}{c} H=\text{MolFormer}(t)\\ =[h_{0},h_{1}\ldots ,h_{i},\ldots ,h_{L}]\in R^{(L+1)\times d_{\text{dim}}}, \end{array}$$

where H denotes the hidden-state matrix, $h_{i}\in R^{d_{\text{dim}}}$ is the hidden vector of the *i*-th token, $d_{\text{dim}}=768$ is the hidden dimension, and *L* is the total sequence length including special tokens and padding. MolFormer utilizes a modified linear attention mechanism with rotary positional embeddings:


(2) $$\text{Attention}(Q,K,V)=\frac{\sum_{n=1}^{N}\langle \varphi (R_{m}q_{m}),\varphi (R_{n}k_{n})\rangle v_{n}}{\sum_{n=1}^{N}\langle \varphi (R_{m}q_{m}),\varphi (R_{n}k_{n})\rangle },$$

where Q,K,V are the query, key, and value matrices, $q_{m}$ is the *m*-th row of Q, $k_{n}$, $v_{n}$ are the *n*-th rows of K and V, $R_{m},R_{n}$ are position-dependent rotary transformation matrices, and φ(·) denotes a random feature mapping function for linear attention approximation. This mechanism provides an effective balance between computational efficiency and expressiveness, capturing both local chemical bonds and long-range dependencies.

Unlike conventional BERT-style models, MolFormer-XL recommends using the mean of all valid token embeddings as the molecule-level representation instead of relying solely on the [CLS] token. Let M denote the attention mask indicating valid tokens ($M_{i}\in \{0,1\}$). The molecule-level embedding is computed by mean pooling over all valid tokens in the last layer:


(3) $$d_{\text{Mol}}=\frac{\sum_{i=0}^{L}M_{i}\cdot h_{i}}{\sum_{i=0}^{L}M_{i}},$$

where the denominator ensures an accurate average over valid tokens, yielding a robust global representation that aggregates all local sequence information.

To better align the pre-trained embeddings with downstream drug synergy prediction, we further refine $d_{\text{Mol}}$ using a lightweight two-layer multilayer perceptron (MLP) with GELU activations in the hidden layer. The resulting projected embedding serves as the final molecular feature for synergy modeling. For a drug pair (A,B), this process produces their respective projected embeddings $d_{A},d_{B}\in R^{128}$, which are then input to the synergy prediction module.

#### 2.1.2 Cell line genomic feature encoding

For cell line *C*, we first collect its 954-dimensional gene expression profile as the raw input. To obtain a compact and informative feature representation, we employ a MLP to progressively abstract the gene expression features. The MLP uses a gradual dimensionality reduction strategy, mapping the high-dimensional input into a lower-dimensional feature space to form the final cell line feature vector $c\in R^{1024}$. After each fully connected layer, layer normalization (Ba *et al.* 2016) is applied to stabilize training, and the GELU (Gaussian Error Linear Unit) (Hendrycks 2016) activation function is used to introduce nonlinear transformation capability, thereby enhancing the model’s representational power. GELU is defined as:


(4) $$\text{GELU}(x)=x\cdot \Phi (x)=x\cdot \frac{1}{2}[1+\text{erf}(\frac{x}{\sqrt{2}})],$$

where Φ(x) denotes the cumulative distribution function of the standard normal distribution, and erf(·) is the error function.

### 2.2 Bidirectional environmental condition modulator

#### 2.2.1 Environmental condition encoder

To accurately model an environmental condition vector composed of cell line and drug features, we propose an Environmental Condition Encoder (ECE). This module takes the cell line representation c and another drug representation d as input, and outputs an environmental condition vector con, providing guidance for subsequent modules.

We first construct an initial joint representation by concatenating multi-perspective interactions:


(5) $$z_{0}=[c,d,c⊙d,c-d]\in R^{4H},$$

where ⊙ denotes the Hadamard product. This initial representation $z_{0}$ is then projected into a lower-dimensional latent space and normalized through a linear layer followed by GELU activation and layer normalization, yielding the representation z.

To enrich the modeling of environmental condition, we design a dual-path interaction structure consisting of a gated nonlinear branch and a low-rank bilinear branch. The gated branch adopts a GEGLU-style structure to enhance nonlinear interactions under environmental conditions (Shazeer 2020). Specifically, two linear projections are first applied to the latent representation z, and the output of one branch is modulated by a GELU-activated gate:


(6) $$z_{\text{gate}}=(W_{a}z)⊙\text{GELU}(W_{g}z),$$

where $W_{a}$ and $W_{g}$ are learnable projection matrices. This formulation follows the generalized gated linear unit (GEGLU) design. Compared with sigmoid-based gating, GEGLU provides smoother gradient propagation and richer activation dynamics, thereby improving representation capacity for complex condition-dependent interactions. The low-rank bilinear branch (Kim *et al.* 2017) explicitly models high-order interactions between cell line and drug features:


(7) $$z_{\mathrm{bilinear}}=\sum_{k=1}^{r}w_{k}(u_{k}^{⊤}c)(p_{k}^{⊤}d),$$

where $u_{k},p_{k}\in R^{H}$ and $w_{k}\in R^{H}$ are learnable parameters, and *r* denotes the rank, set as r=max(16,H/8). This formulation corresponds to a low-rank factorization of a full bilinear interaction tensor, enabling expressive modeling of cross-feature dependencies while controlling parameter complexity.

The enhanced features are aggregated via residual fusion and further refined through channel-wise adaptive recalibration to obtain the environmental condition vector con:


(8) $$z^{*}=z+z_{\mathrm{gate}}+z_{\mathrm{bilinear}},$$

(9) $$\mathrm{con}=z^{*}⊙R(z^{*}),$$

where R(·) denotes a channel-wise feature recalibration function implemented with Squeeze-and-Excitation (SE) (Hu *et al.* 2018). Specifically, an adaptive weight vector $s\in {\left(0,1\right)}^{H}$ is generated for each channel and applied through element-wise multiplication:


(10) $$R(z^{*})=\sigma (W_{2}\text{SiLU}(W_{1}z^{*})),$$

where $W_{1}$ and $W_{2}$ are learnable parameters, and σ denotes the sigmoid activation function.

#### 2.2.2 Residual feature-wise linear modulation

To enable deep interactions among drug-drug-cell triplets at the feature level, we propose a Residual Feature-wise Linear Modulation (R-FiLM) (Perez *et al.* 2018), which introduces residual connections (He *et al.* 2016) into the FiLM framework to improve the stability and expressive capacity of feature modulation. R-FiLM adaptively modulates drug features using environmental condition vectors, such that each drug representation not only encodes its intrinsic properties but also reflects its potential functional patterns under specific drug combinations and cellular conditions.

Specifically, for drug *A*, its environmental condition vector $\mathrm{co}n_{A}$ is generated from the cell line features c and drug *B*’s features $d_{B}$ via the Environmental Condition Encoder (ECE). Symmetrically, the environmental condition vector for drug *B*, $\mathrm{co}n_{B}$, is generated from the cell line features and drug *A*’s features $d_{A}$ through the same process.

Subsequently, the drug features are modulated through the R-FiLM module:


(11) film=d+FiLM(d,con)=d+γ⊙d+β,

here, γ and β denote the scaling and bias parameters derived from the environmental condition via an MLP and channel-wise partitioning. We specifically constrain γ using the tanh function (range [−1,1]) to enable bidirectional modulation, allowing for both the amplification and suppression of drug features while preventing feature divergence to ensure training stability. The modulated features are then normalized to obtain the final drug representations, $\mathrm{fil}m_{A}$ and $\mathrm{fil}m_{B}$, for downstream interaction prediction tasks.

#### 2.2.3 Drug pair feature fusion

After modulating the features, we simulate the interaction patterns between drug pairs under the current specific environment. For each drug pair, this module employs a gating mechanism to fuse two sources of information: complex interaction patterns extracted via nonlinear transformations, and inter-drug feature differences.

First, the gating signal *g* is computed as:


(12) $$g=\sigma (W_{g}\cdot [\mathrm{fil}m_{A},\mathrm{fil}m_{B}]),$$

where σ denotes the Sigmoid activation function, $W_{g}$ is a learnable weight matrix, and $\left[\mathrm{fil}m_{A},\mathrm{fil}m_{B}\right]$ represents the concatenation of the modulated features of drugs *A* and *B*. The gating signal *g* controls the model’s attention to different sources of information.

Next, the nonlinear interaction features *t* are extracted as:


(13) $$t=\text{GELU}(W_{t}\cdot [\mathrm{fil}m_{A},\mathrm{fil}m_{B}]),$$

where $W_{t}$ is a learnable weight matrix, and GELU is the Gaussian Error Linear Unit used to capture complex nonlinear interaction patterns.

Finally, the gating signal is used to fuse the two sources of information:


(14) $$I_{\mathrm{film}}=g⊙t+(1-g)⊙|\mathrm{fil}m_{A}-\mathrm{fil}m_{B}|,$$

where ⊙ denotes element-wise multiplication, and $\left|\mathrm{fil}m_{A}-\mathrm{fil}m_{B}\right|$ is the feature difference vector, preserving contrastive information between the drug pair.

#### 2.2.4 Predictor

To dynamically simulate the interaction patterns of drug pairs, we first obtain the fused interaction features of the modulated drug pair $\left(\mathrm{fil}m_{A},\mathrm{fil}m_{B}\right)$, denoted as $I_{\mathrm{film}}$, and the fused interaction features of the initial drug pair $\left(d_{A},d_{B}\right)$, denoted as I, to capture the feature changes before and after interaction.

Subsequently, the four types of information $\mathrm{fil}m_{A}$, $\mathrm{fil}m_{B}$, $I_{\mathrm{film}}$ and I are concatenated to form a comprehensive feature vector $E_{\mathrm{concat}}\in R^{4H}$ incorporating both environmental and interaction information. To predict drug synergy, $E_{\mathrm{concat}}$ is fed into a MLP to obtain the predicted probability:


(15) $$\hat{y}=\sigma (W_{o}\cdot {\text{MLP}}_{\mathrm{head}}(E_{\mathrm{concat}})),$$

where ${\text{MLP}}_{\mathrm{head}}(\cdot )$ represents the MLP network, $W_{o}$ is the output weight matrix, σ(·) denotes the Sigmoid activation function, and $\hat{y}\in [0,1]$ indicates the predicted probability of synergy for the drug pair.

The model is trained using the binary cross-entropy (BCE) loss:


(16) $$L_{\mathrm{BCE}}=-\frac{1}{N}\sum_{i=1}^{N}\;[y_{i}\;\text{log}\;{\hat{y}}_{i}+(1-y_{i})\;\text{log}(1-{\hat{y}}_{i})],$$

where *N* is the number of samples, $y_{i}\in \{0,1\}$ denotes the ground-truth label, and ${\hat{y}}_{i}$ is the predicted probability.

## 3 Results

### 3.1 Dataset

The benchmark dataset used in this study is derived from the O’Neil *et al.* (2016) high-throughput screening study, which evaluated 22 737 drug combination experiments across 39 cancer cell lines using 38 compounds. Drug synergy is quantified using the Loewe model (Loewe 1953), a reference-based approach that defines synergy as deviation from additivity. Specifically, synergy scores are calculated from 4 by 4 dose–response matrices using the Combenefit tool. Following the preprocessing strategy of DeepDDS, combinations with Loewe scores >10 are labeled as synergistic, while those with scores below 0 are labeled as antagonistic. This threshold is chosen to mitigate the influence of near-zero additive combinations and improve classification robustness. After filtering, the final dataset comprises 12 415 unique drug pair–cell line combinations, involving 36 drugs and 31 cancer cell lines, resulting in a balanced dataset. Drug SMILES representations are obtained from the DrugBank database (Wishart *et al.* 2018) to encode molecular structures. Gene expression profiles of cell lines are derived from the Cancer Cell Line Encyclopedia (CCLE) (Barretina *et al.* 2012) and intersected with the “Landmark” gene set of the LINCS project (Subramanian *et al.* 2017), which contains 1000 representative genes. After gene annotation and removal of redundant features, 954 representative genes are finally selected to characterize each cell line.

To further evaluate model generalization on out-of-distribution data, the AstraZeneca dataset (Menden *et al.* 2019) is employed as an independent test set. This dataset contains molecular features of 118 drugs and 85 cancer cell lines. To prevent overlap with the training data, drugs and cell lines shared with the O’Neil dataset are removed, resulting in 668 drug combination samples covering 57 drugs and 21 cancer cell lines. The Loewe score is used as the synergy metric.

### 3.2 Baselines

To evaluate the effectiveness of the proposed method in drug synergy prediction, we select 12 representative baseline models, including seven recently proposed deep learning models MFSynDCP (Dong *et al.* 2024), CFSSynergy (Rafiei *et al.* 2024), DFFNDDS (Xu *et al.* 2023), DeepTraSynergy (Rafiei *et al.* 2023), DeepDDS (Wang *et al.* 2022), GraphSynergy (Yang *et al.* 2021), and DeepSynergy (Preuer *et al.* 2018), as well as five classical machine learning methods XGBoost, GBM, AdaBoost, SVM, and MLP (see Section 1, available as supplementary data at *Bioinformatics* online, for details). All models are systematically evaluated using seven performance metrics, including the area under the receiver operating characteristic curve (AUROC), the area under the precision-recall curve (AUPRC), accuracy (ACC), balanced accuracy (BACC), precision (PREC), recall, and Cohen’s kappa (KAPPA), to ensure the comprehensiveness and reliability of the comparisons. Detailed configurations of the experimental parameters are provided in Section 2 and Table A1, available as supplementary data at *Bioinformatics* online.

**Table 1:** Results of the five-fold cross-validation on the O’Neil dataset under transductive settings.^a^

	AUROC	AUPRC	ACC	BACC	Precision	Recall	Kappa
Env-Syn	**0.95 ± 0.01**	**0.95 ± 0.01**	**0.88 ± 0.01**	**0.88 ± 0.01**	**0.87 ± 0.02**	**0.88 ± 0.01**	**0.76 ± 0.01**
MFSynDCP	0.92 ± 0.01	0.92 ± 0.01	0.85 ± 0.07	0.85 ± 0.01	0.86 ± 0.01	0.86 ± 0.01	0.70 ± 0.01
CFSSynergy	0.90 ± 0.01	0.90 ± 0.02	0.82 ± 0.02	0.82 ± 0.02	0.82 ± 0.03	0.81 ± 0.01	0.61 ± 0.05
DFFNDDS	0.93 ± 0.01	0.92 ± 0.01	0.86 ± 0.01	0.86 ± 0.01	0.85 ± 0.01	0.86 ± 0.03	0.72 ± 0.03
DeepTraSynergy	0.88 ± 0.02	0.88 ± 0.02	0.78 ± 0.02	0.78 ± 0.02	0.81 ± 0.02	0.75 ± 0.02	0.54 ± 0.04
DeepDDS	0.93 ± 0.01	0.93 ± 0.01	0.85 ± 0.01	0.85 ± 0.07	0.85 ± 0.07	0.85 ± 0.07	0.71 ± 0.21
GraphSynergy	0.91 ± 0.01	0.90 ± 0.01	0.83 ± 0.01	0.83 ± 0.01	0.84 ± 0.01	0.80 ± 0.01	0.64 ± 0.01
DeepSynergy	0.88 ± 0.01	0.87 ± 0.01	0.80 ± 0.01	0.80 ± 0.01	0.81 ± 0.01	0.75 ± 0.01	0.59 ± 0.05
XGBoost	0.92 ± 0.01	0.92 ± 0.01	0.83 ± 0.01	0.83 ± 0.01	0.84 ± 0.01	0.84 ± 0.01	0.68 ± 0.01
GBM	0.85 ± 0.02	0.85 ± 0.01	0.76 ± 0.02	0.76 ± 0.02	0.77 ± 0.01	0.74 ± 0.01	0.53 ± 0.04
Adaboost	0.83 ± 0.01	0.83 ± 0.03	0.74 ± 0.01	0.74 ± 0.02	0.74 ± 0.02	0.72 ± 0.01	0.48 ± 0.03
SVM	0.58 ± 0.01	0.56 ± 0.02	0.54 ± 0.01	0.54 ± 0.01	0.54 ± 0.01	0.51 ± 0.12	0.08 ± 0.04
MLP	0.65 ± 0.02	0.63 ± 0.05	0.56 ± 0.06	0.56 ± 0.05	0.54 ± 0.04	0.53 ± 0.22	0.12 ± 0.04

a Bold indicates the best results, and underlines indicate the second-best performance.

### 3.3 Transductive setting

We conduct performance comparison experiments using five-fold cross-validation. As summarized in Table 1, our proposed model consistently achieves the best performance across all seven evaluation metrics. Notably, the AUROC and AUPRC both reach 0.95±0.01, simultaneously surpassing the 0.95 threshold, indicating excellent discriminative capability as well as strong practical utility in classification tasks. Furthermore, the Kappa achieves 0.76±0.01, representing a 5.56% improvement over the second-best model, DFFNDDS (Xu *et al.* 2023). Since Kappa reflects the agreement between predicted labels and ground truth, this improvement indicates that the proposed model yields more robust and stable classification decisions.

When compared with traditional machine learning approaches, our model demonstrates substantial improvements, with ACC and BACC increasing by at least 6.02% over the best-performing baseline, and Recall improving by no <4.76%. These results highlight the superior capability of our method in capturing complex data patterns, extracting discriminative features, and performing effective classification. In addition, the proposed model consistently achieves the highest precision among all evaluated methods, further validating the advantages conferred by its architectural design and training strategy in enhancing classification accuracy.

### 3.4 Inductive setting

#### 3.4.1 Performance evaluation on LOOCV

**Figure 2:**
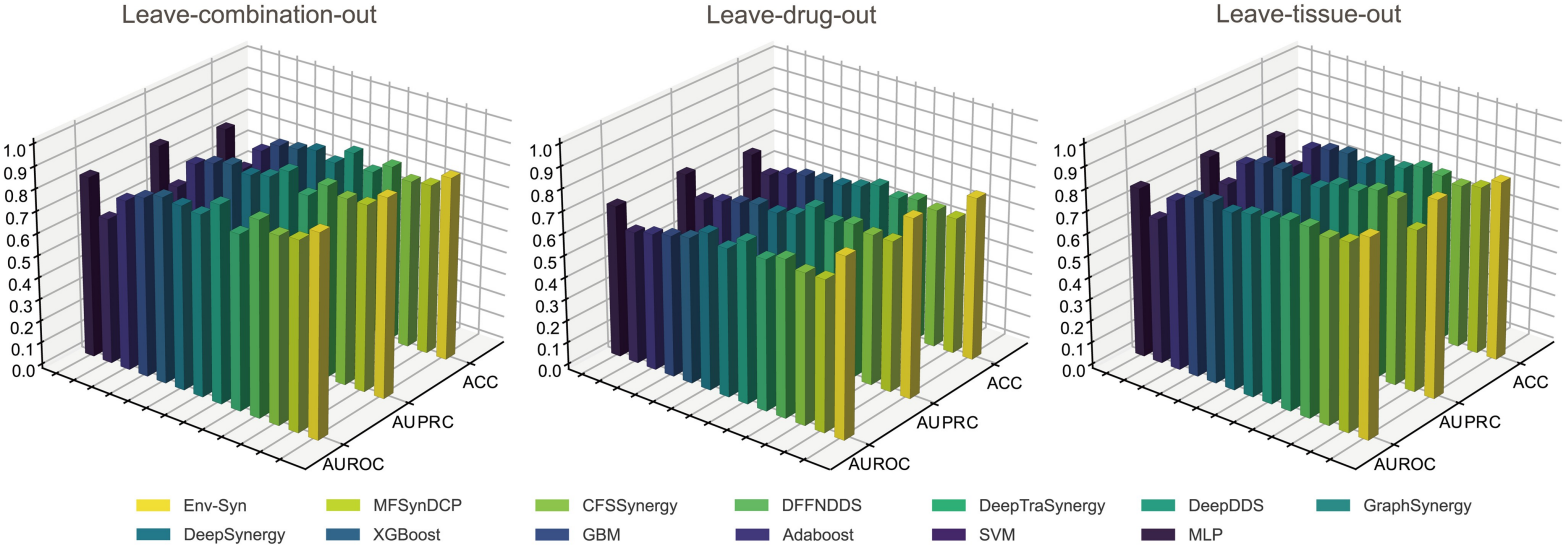
Results of Leave-One-Out Cross-Validation under three evaluation scenarios.

We further evaluate the performance of our model under three Leave-one-out scenarios (see Section 3, available as supplementary data at *Bioinformatics* online, for details). As shown in Fig. 2, in the Leave-drug-out setting, our model achieves AUROC and AUPRC values exceeding 0.81. Compared with the second-best baseline, it exhibits an improvement of ∼11% in AUROC and 12.5% in AUPRC, demonstrating a clear advantage. In the Leave-combination-out scenario, AUROC reaches a peak of 0.91, and in the Leave-tissue-out scenario, ACC surpasses 0.7, reaching 0.74, both indicating robust predictive performance on previously unseen combination and tissue level data.

Across all three Leave-one-out scenarios, our model outperforms all other methods, with an average improvement of 5%–15% across all metrics, validating its predictive capability in novel scenarios.

#### 3.4.2 Independent experimental validation

**Figure 3:**
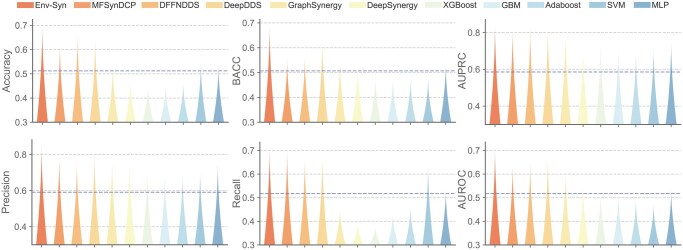
Results on the independent test dataset under inductive settings.

To rigorously assess the model’s generalization to out-of-distribution data, we trained Env-Syn on the O’Neil dataset and evaluated it on an independent test set provided by AstraZeneca. As shown in Fig. 3, Env-Syn consistently outperforms existing approaches across all metrics, with the corresponding quantitative results summarized in Table A3, available as supplementary data at *Bioinformatics* online. It achieves an AUROC of 0.70, exceeding the 0.66 and 0.64 achieved by DeepDDS and DFFNDDS, respectively, as well as the performance of conventional machine learning models. Notably, Env-Syn maintains superior and stable performance in recall, demonstrating reliable classification consistency and strong recognition of positive cases. These results indicate that Env-Syn effectively captures the interaction patterns between drugs and cell lines, rather than overfitting to the specific distribution.

### 3.5 Ablation study

#### 3.5.1 Ablation study on model components

**Figure 4:**
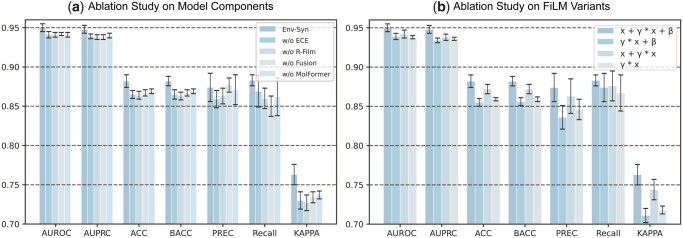
Ablation studies on model components and FiLM variants. (a) Performance comparison after removing individual modules. (b) Performance comparison among different FiLM formulations.

To assess the contribution of each component in our model, we conduct four ablation experiments (see Section 5.1, available as supplementary data at *Bioinformatics* online, for details). As shown in Fig. 4a, removing any component leads to consistent performance decay. The most significant declines occur in the w/o ECE and w/o R-FiLM variants, where Kappa drops by 4.33% to 0.730 and 4.72% to 0.727, respectively. Mechanistically, ECE serves as the context extractor that distills cell-drug environmental cues, while R-FiLM acts as a dynamic bridge that projects these cues onto drug representations via affine transformations. Without these modules, the model reverts to a static background assumption, failing to capture the environment-specific synergistic triggers that vary across different cell lines.

Moreover, the w/o MolFormer variant confirms that while Env-Syn remains robust when using traditional similarity features, the semantic richness of MolFormer embeddings is vital for capturing high-dimensional chemical contexts, as its removal results in an AUROC decrease of 0.84% to 0.942 and a Kappa decrease of 3.41% to 0.737.

#### 3.5.2 Ablation study on FiLM variants

Meanwhile, we perform ablation experiments on three different FiLM variants (see Section 5.2, available as supplementary data at *Bioinformatics* online, for details) to analyze how different forms of environment-conditioned modulation affect model performance. As illustrated in Fig. 4b, the full R-FiLM structure (x+γ·x+β) achieves the optimal performance with a Kappa of 0.763 and an AUPRC of 0.948.

The standard FiLM formulation (Perez *et al.* 2018), which lacks the residual shortcut, results in a significant performance decay, with Kappa dropping by 6.82% to 0.711 and AUPRC decreasing to 0.934. This decline suggests that aggressive affine transformations without identity preservation may attenuate the drug’s intrinsic chemical signatures. Furthermore, removing the bias term, as demonstrated by the x+γ·x variant, leads to a Kappa decrease of 2.49% to 0.744, highlighting the necessity of translation-based modulation for capturing the shifting baseline of drug efficacy across diverse cellular environments. The variant utilizing only scaling exhibits a 5.90% drop in Kappa to 0.718, further confirming that a simple scaling factor is insufficient for modeling complex ternary interactions. These results demonstrate that R-FiLM effectively balances the preservation of inherent molecular features with the incorporation of expressive, environment-conditioned signals.

#### 3.5.3 Symmetry analysis of drug combinations

**Figure 5:**
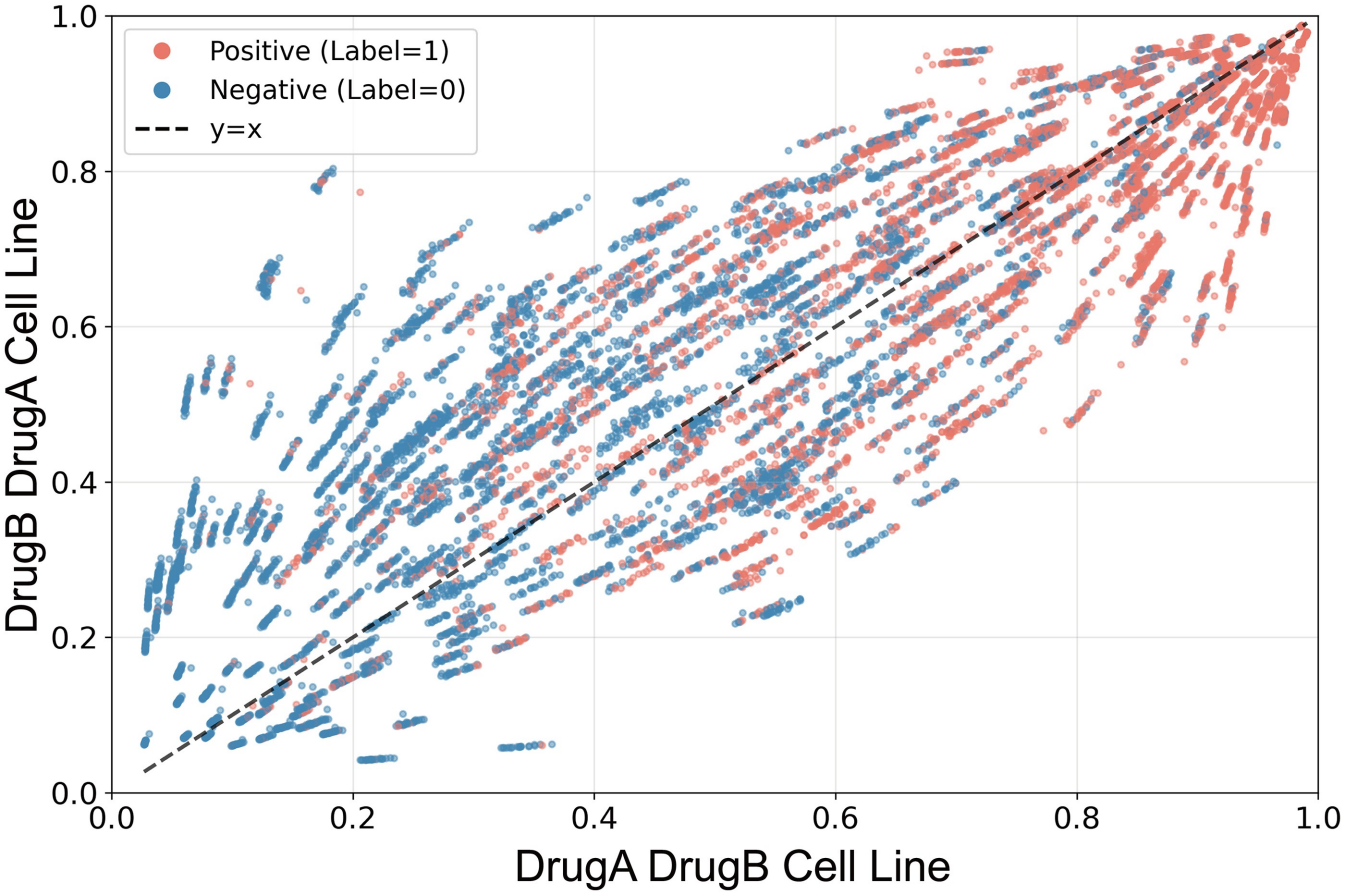
Scatter plot of predicted synergy scores under symmetric drug input orders.

To assess the robustness of our framework regarding the sequence of inputs, we conducted a systematic symmetry analysis by evaluating the model’s sensitivity to drug ordering. For each triplet (Drug A, Drug B, Cell Line), we construct its symmetric triplet (Drug B, Drug A, Cell Line) and obtain the corresponding predictions. As shown in Fig. 5, the predicted values for all samples are highly linearly correlated with those of their symmetric counterparts, with a PCC of 0.892, indicating that the model is insensitive to the order of drug inputs.

#### 3.5.4 Dimensionality reduction visualization

**Figure 6:**
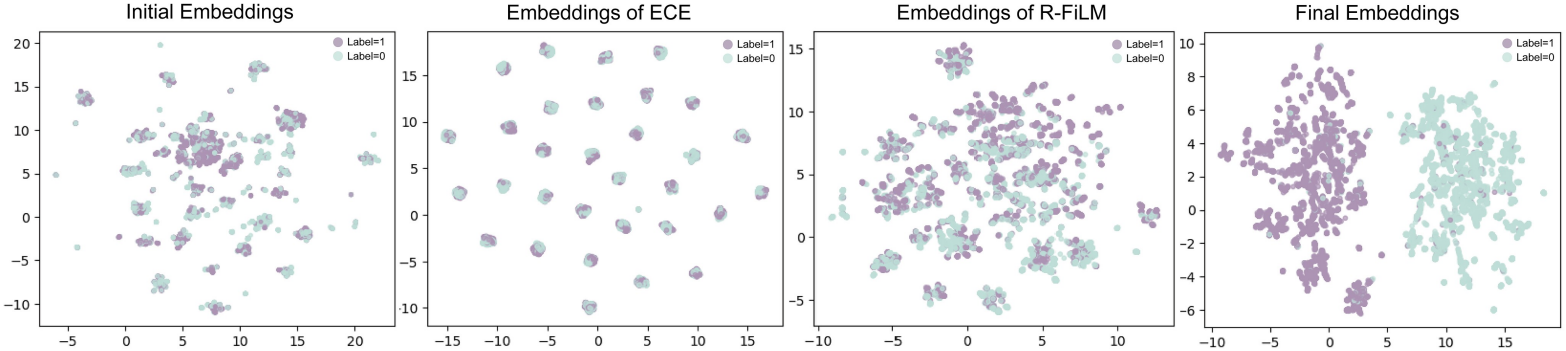
UMAP visualization of feature representations at different stages of the model.

To systematically assess the discriminative capacity of features at different stages of the model, we employ Uniform Manifold Approximation and Projection (UMAP) (McInnes *et al.* 2018) to perform low-dimensional visualization of the input representations, intermediate features, and final outputs. As shown in Fig. 6, at the initial stage, samples exhibit a largely unstructured distribution in the low-dimensional embedding space. After incorporating the environmental condition vector, the feature embeddings begin to reveal localized patterns. Following the R-FiLM module, the representations show an emerging trend of separation. At the final stage, the embeddings demonstrate clear and well-defined class separability.

#### 3.5.5 Novel drug combination prediction

The experiment employs a random recombination strategy to construct the test set, strictly adhering to a sample deduplication principle to ensure that it consists exclusively of “novel drug combination–cell line” pairs that the model has not encountered during training. Using the benchmark dataset as the training set, we predict the synergy probabilities for these new combinations. HT-29 (colorectal adenocarcinoma), A2780 (ovarian carcinoma), and KPL-1 (breast cancer) cell lines are selected as representative models for detailed analysis due to their high data density in the benchmark dataset and their well-characterized roles in prevalent solid tumors. (see Section 6, available as supplementary data at *Bioinformatics* online, for details of the cell lines). Table 2 presents the top five predicted combinations for each cell line along with their corresponding literature validation.

**Table 2:** Top five predicted novel synergistic drug combinations for three cell lines, with literature validation.^a^

Cell line	Drug1	Drug2	PMID and reference
HT-29	DASATINIB	METFORMIN	34421608 (Zhu *et al.* 2021)
	TOPOTECAN	MK-2206	37521473^b^ (Gao *et al.* 2023)
	DINACICLIB	BEZ-235	27529512 (Beale *et al.* 2016)
	L778123	METFORMIN	32911743 (Seo *et al.* 2020)
	TOPOTECAN	ZOLINZA	37092854^b^ (Madkour *et al.* 2023)
A2780	ZOLINZA	MK-2206	NA
	DOXORUBICIN	MK-2206	20571069 ( Hirai *et al.* 2010)
	DINACICLIB	MK-2206	27663592^b^ (Au-Yeung *et al.* 2017)
	MK-8776	TOPOTECAN	25884494 ( Kim *et al.* 2015)
	MK-8669	METFORMIN	28122439 (Liu *et al.* 2016)
KPL-1	TOPOTECAN	PACLITAXEL	14529450 (Rudolf and Cervinka, 2003)
	DEXAMETHASONE	PACLITAXEL	32240873^b^ (Diab *et al.* 2020)
	PD325901	PACLITAXEL	11038347 (MacKeigan *et al.* 2000)
	DOXORUBICIN	CYCLOPHOSPHAMIDE	33491966^b^ (Kuchira *et al.* 2021)
	DOXORUBICIN	5-FU	16168113^b^ (Zoli *et al.* 2005)

a Underlines and

b denote drug combinations demonstrating synergistic effects at the cell line and tissue levels, respectively. NA indicates a lack of supporting literature, while unmarked entries signify synergistic effects of the corresponding inhibitors within specific tissues.

From the underlined references in Table 2, for the A2780 cell line, Hirai *et al.* (2010) demonstrate that the primary mechanism of MK-2206 is the precise inhibition of the Akt pathway, thereby eliminating the survival advantage of tumor cells and producing a synergistic effect with DOXORUBICIN, ultimately suppressing tumor growth through enhanced apoptosis (Hirai *et al.* 2010). Concurrently, Kim *et al.* (2015) show that MK-8776-mediated inhibition of CHEK1 effectively blocks DNA repair, significantly enhancing the cytotoxicity of Topotecan against A2780 cells (Kim *et al.* 2015).

The mechanisms of certain drugs have been characterized in the corresponding disease context. For example, in the ABCG2-overexpressing, drug-resistant colorectal cancer S1-M1-80 cell line, MK-2206 competitively binds to the substrate-binding site of the ABCG2 transporter and stimulates its ATPase activity, thereby effectively blocking the efflux of Topotecan mediated by this protein (Gao *et al.* 2023).

For novel drug combinations lacking direct experimental validation, we perform mechanistic inference. For instance, the combination of PACLITAXEL with a MEK inhibitor exhibits substantial predicted synergistic potential in breast cancer by blocking the PACLITAXEL-induced compensatory MEK/ERK survival signaling (MacKeigan *et al.* 2000). Given that PD325901 is a potent and highly selective MEK inhibitor capable of effectively disrupting this pathway, it can be inferred that the combination of PD325901 and PACLITAXEL possesses synergistic potential through the same mechanism.

## 4 Discussion and conclusion

In this study, we propose Env-Syn, an environment-aware framework for drug synergy prediction based on bidirectional environmental conditional encoding and feature modulation. By incorporating environment aware R-FiLM and a gated interaction fusion mechanism, Env-Syn effectively captures synergistic effects of drug combinations under specific cellular contexts.

Extensive experiments demonstrate that Env-Syn consistently outperforms existing methods across multiple datasets and evaluation settings. Importantly, the model maintains strong predictive performance on unseen drugs, combinations, and tissue contexts, as well as on independent out-of-distribution datasets, highlighting its robust generalization capability and effective learning of drug-drug-cell environment interactions. Visualization analyses further reveal that representations of synergistic and non-synergistic samples become increasingly separable during training, indicating effective feature disentanglement. Among 15 predicted novel synergistic drug combinations, eight are directly validated, highlighting the practical utility of Env-Syn for combination drug discovery.

Despite its strong performance, several limitations should be acknowledged. First, drugs are represented using 1D SMILES sequences through MolFormer-XL, which may lack 3D conformational and stereochemical information important for certain molecular interactions. Second, Env-Syn currently models drug pairs, whereas clinical treatments often involve three or more drugs. Nevertheless, the modular design of the ECE and R-FiLM components provides a scalable basis for modeling higher-order combinations. Third, since the cellular environment is primarily characterized by gene expression profiles, future work may integrate additional environmental information, such as knowledge graphs or gene regulatory networks, to construct more comprehensive cellular context representations and further improve predictive accuracy. Finally, developing user-friendly web interfaces is one of our key areas of focus for future work. This would facilitate use by researchers without programming experience and support broader exploration of potential drug synergy through computational prediction.

Overall, Env-Syn provides an effective and flexible framework for drug synergy prediction, with promising applications in combination therapy screening and precision treatment design.

## Supplementary Material

btag256_Supplementary_Data

## Data Availability

Data and source code supporting this study are openly available at GitHub: https://github.com/AnQi-87/Env-Syn. A persistent snapshot of the code used in this study has been archived on Figshare with the DOI: 10.6084/m9.figshare.31832731.
