# Supplementary material for: Learning drug synergy through environment-conditioned feature modulation
Source: Bioinformatics. 2026 May 5;42(5):btag256. doi: 10.1093/bioinformatics/btag256 (PMC13202328; doi:10.1093/bioinformatics/btag256)
Supplement: btag256_Supplementary_Data [file btag256_supplementary_data.pdf]

Supplementary Material

1 Baseline

This study selects 12 representative benchmark models, including seven deep learning methods proposed in recent years—MFSynDCP, CFSSynergy, DFFNDDS, DeepTraSynergy, DeepDDS, GraphSynergy, and DeepSynergy—as well as five traditional machine learning methods: XGBoost (Extreme Gradient Boosting), GBM (Gradient Boosting Machine), AdaBoost (Adaptive Boosting), SVM (Support Vector Machine) and MLP (Multi-Layer Perceptron).

The following section provides a description of the seven deep learning models:

- MFSynDCP: This model utilizes a deep Graph Attention Network (GAT) to learn high-dimensional drug features from molecular graphs and a MLP to extract genomic features from cancer cell lines. It introduces an adaptive attention mechanism for graph aggregation to capture critical drug substructures and a multi-source feature interactive learning controller with a gating structure to regulate information transfer and fusion between heterogeneous data sources. By integrating these features, the model predicts the synergistic effects of drug combinations.

- CFSSynergy: This model proposes a hybrid approach that integrates feature-based and similarity-based viewpoints for drug synergy prediction. It employs a transformer-based architecture to extract discriminative representations for drugs and utilizes the Node2Vec algorithm to create a protein-protein similarity matrix for cell line feature enhancement. By computing and combining the similarities between unique drugs and cell lines with their learned features, the model constructs a comprehensive representation. These multi-view features are finally fed into an XGBoost regressor to predict synergistic interactions.

- DFFNDDS: The model predicts synergistic drug combinations by integrating a fine-tuned pre-trained language model with a dual feature fusion mechanism. It employs a BERT model fine-tuned via SimCSE to extract high-quality vector representations from drug SMILES sequences. To capture complex interactions between drug features and cell line gene expressions, the model utilizes a dual feature fusion mechanism that operates at both the bit-wise level using a highway network and the vector-wise level using a multi-head attention mechanism. The final concatenated features are passed through a predictor module to output the synergy score.

- DeepTraSynergy: This model is a deep learning-based multitask framework that predicts drug combination synergy by integrating multimodal data, including drug-target, protein-protein, and cell-target interactions. It utilizes a Transformer-based architecture to learn discriminative drug feature representations. Notably, the model enhances synergy prediction by incorporating two auxiliary tasks: predicting drug-protein interactions and toxic effects. By jointly optimizing three corresponding loss functions (synergy, toxic, and interaction loss), the framework leverages high-order biological relationships, such as PPI networks, to capture complex pharmacological mechanisms and improve overall prediction accuracy.

- DeepDDS: This model presents a deep learning framework based on graph neural networks (GNN) and attention mechanisms to identify synergistic drug combinations for specific cancer cell lines. DeepDDS utilizes either a Graph Convolutional Network (GCN) or a GAT to extract feature embeddings from drug molecular structures, where atoms are represented as nodes and chemical bonds as edges. Simultaneously, the genomic features of cancer cell lines are encoded using a MLP from gene expression profiles. By concatenating the drug and cell line embeddings, the model employs multiple fully connected layers to perform binary classification, predicting whether a drug pair is synergistic or antagonistic.

- GraphSynergy: This model is an end-to-end deep learning framework that leverages protein-protein interaction (PPI) networks to predict synergistic drug combinations. It employs a spatial-based GCN with an attention mechanism to encode the high-order topological relationships between protein modules targeted by drugs and those associated with specific cancer cell lines. By explicitly calculating therapy scores (similarity between drug pairs and cell lines) and toxicity scores (similarity between the two drugs), the model evaluates the pharmacological effects to predict synergy.

- DeepSynergy: The model utilizes a deep feed-forward neural network to predict the synergy of anti-cancer drug combinations. It integrates chemical information of drug pairs (e.g., ECFP_6 fingerprints, physico-chemical properties, and toxicophores) with genomic profiles of cell lines (gene expression data) as input features. By modeling the non-linear interactions between drug and cell line representations through multiple hidden layers (including conic and rectangular architectures), the model learns a unified representation to accurately estimate the synergy scores for novel drug-pair cell line combinations.

2 Hyperparameter settings

Our experimental setup employs 5-fold cross-validation, with a training-to-test split ratio of 4:1 for each fold. The final hyperparameter configurations for Env-Syn are determined based on the model's empirical performance on the validation set. Table A1 summarizes the considered candidate values, with the optimal settings highlighted in bold. Specifically, we use a learning rate of 1e-4, a dropout rate of 0.2, a batch size of 1024, a hidden dimension of 1024, train the model for 50 epochs, and set the random seed to 2023 to achieve a balance between convergence and computational efficiency.

Table A1: Hyperparameter settings for Env-Syn. The optimal configurations are highlighted in bold.

Hyperparameter	Values considered
Learning rate	5e-5; 7e-5; **1e-4**; 3e-4; 5e-4
Dropout rate	0.1; **0.2**; 0.3; 0.4; 0.5
Batch size	64; 128; 256; 512; **1024**
Epochs	30; **50**; 100; 150; 200
Hidden dim	64; 128; 256; 512; **1024**
Random seed	2022; **2023**; 2024; 2025; 2026

3 Leave-One-Out Cross Validation scenarios setting

The Leave-One-Out Cross Validation (LOOCV) is designed to evaluate the model's generalization performance on unseen drug combinations, individual drugs, or cell lines. Based on these three criteria, three LOOCV modes are established: Leave-combination-out, Leave-drug-out, and Leave-tissue-out. The detailed descriptions of these settings are as follows:

- **Leave-combination-out**: Simulates the synergy prediction of "unseen drug combinations" in a given cell line.
- **Leave-drug-out**: Simulates the synergy prediction of a "newly introduced single drug" in combination with existing drugs across known cell lines.
- **Leave-tissue-out**: Simulates the synergy prediction of known drug combinations within a "novel tissue" environment.

We further supplement the results of the LOOCV in Table A2, providing a comprehensive evaluation of the model's performance under various generalization settings.

Table A2: Results of the LOOCV on the O'Neil dataset under inductive settings.

	Leave-combination-out			Leave-drug-out			Leave-tissue-out		
	ROC AUC	PR AUC	ACC	ROC AUC	PR AUC	ACC	ROC AUC	PR AUC	ACC
Env-Syn	0.91±0.01	0.90±0.02	0.83±0.02	0.81±0.05	0.81±0.05	0.74±0.04	0.89±0.03	0.89±0.03	0.81±0.03
MFSynDCP	0.85±0.03	0.84±0.06	0.77±0.08	0.68±0.02	0.68±0.05	0.62±0.05	0.84±0.03	0.73±0.04	0.76±0.07
CFSSynergy	0.84±0.03	0.84±0.03	0.76±0.04	0.68±0.05	0.68±0.06	0.63±0.06	0.83±0.10	0.84±0.06	0.74±0.05
DFFNDDS	0.88±0.01	0.87±0.03	0.80±0.01	0.71±0.05	0.70±0.03	0.65±0.05	0.85±0.04	0.85±0.3	0.76±0.04
DeepTraSynergy	0.79±0.08	0.80±0.09	0.75±0.06	0.62±0.06	0.64±0.10	0.54±0.13	0.85±0.10	0.82±0.11	0.77±0.011
DeepDDS	0.89±0.02	0.88±0.06	0.81±0.03	0.73±0.01	0.72±0.05	0.66±0.02	0.83±0.04	0.82±0.4	0.74±0.03
GraphSynergy	0.82±0.02	0.83±0.02	0.74±0.03	0.67±0.11	0.66±0.06	0.63±0.11	0.82±0.12	0.78±0.11	0.75±0.07
DeepSynergy	0.83±0.03	0.81±0.05	0.77±0.03	0.71±0.07	0.64±0.06	0.61±0.07	0.80±0.01	0.79±0.04	0.71±0.05
XGBoost	0.84±0.02	0.83±0.04	0.75±0.02	0.66±0.09	0.65±0.06	0.61±0.06	0.82±0.01	0.81±0.01	0.73±0.01
GBM	0.81±0.03	0.81±0.04	0.74±0.02	0.64±0.09	0.63±0.09	0.60±0.06	0.81±0.08	0.81±0.05	0.72±0.06
Adaboost	0.77±0.02	0.78±0.02	0.69±0.03	0.62±0.11	0.61±0.06	0.58±0.11	0.77±0.12	0.78±0.11	0.70±0.11
SVM	0.66±0.01	0.65±0.05	0.58±0.01	0.60±0.02	0.59±0.05	0.55±0.03	0.66±0.04	0.66±0.07	0.59±0.05
MLP	0.82±0.03	0.81±0.05	0.74±0.02	0.69±0.05	0.68±0.04	0.62±0.06	0.77±0.07	0.76±0.05	0.70±0.06

4 Independent Experimental Validation

Table A3: Results of the Independent Experimental Validation under inductive settings.

	ROC AUC	PR AUC	ACC	BACC	Precision	Recall	Kappa
Env-Sy	0.70±0.04	0.83±0.07	0.69±0.03	0.68±0.09	0.84±0.06	0.70±0.08	0.32±0.16
MFSynDCP	0.63±0.02	0.81±0.01	0.61±0.15	0.55±0.03	0.78±0.07	0.69±0.34	0.11±0.06
DFFNDDS	0.64±0.11	0.82±0.06	0.66±0.04	0.56±0.10	0.75±0.08	0.66±0.13	0.12±0.13
DeepDDS	0.66±0.12	0.82±0.15	0.64±0.15	0.62±0.13	0.80±0.11	0.67±0.12	0.21±0.29
GraphSynergy	0.61±0.12	0.80±0.11	0.53±0.11	0.55±0.07	0.76±0.10	0.45±0.21	0.09±0.13
DeepSynergy	0.55±0.15	0.71±0.13	0.47±0.14	0.53±0.13	0.75±0.14	0.39±0.17	0.04±0.15
XGBoost	0.52±0.11	0.73±0.12	0.45±0.15	0.49±0.11	0.71±0.09	0.38±0.17	-0.01±0.14
GBM	0.51±0.10	0.71±0.09	0.45±0.12	0.47±0.08	0.69±0.14	0.43±0.12	-0.03±0.14
Adaboost	0.49±0.09	0.69±0.14	0.46±0.17	0.47±0.12	0.69±0.14	0.46±0.15	-0.05±0.17
SVM	0.47±0.11	0.71±0.13	0.54±0.13	0.47±0.15	0.70±0.13	0.63±0.11	-0.04±0.15
MLP	0.53±0.13	0.74±0.12	0.53±0.15	0.53±0.15	0.74±0.13	0.53±0.13	0.05±0.11

5 Ablation study

5.1 Ablation study on model components

To verify the effectiveness of each module in our model, we design three types of ablation experiments, detailed as follows:

- **Env-Syn w/o ECE**: Removal of the Environmental Condition Encoder module. This configuration evaluates the effects of drug pairs without perceiving specific environmental contexts.
- **Env-Syn w/o R-FiLM**: Removal of the Residual Feature-wise Linear Modulation (R-FiLM) module.
- **Env-Syn w/o Fusion**: Removal of the Drug Pair Feature Fusion module. This variant validates the impact of drug pairs when their interaction is not explicitly simulated.

5.2 Ablation study on FiLM variants

To further investigate the internal mechanisms of the module, we conduct intra-module ablation studies using three distinct formulations:

- $\gamma \cdot x + \beta$: The original FiLM operation without the residual connection.
- $x + \gamma \cdot x$: The formulation where the bias term β is removed.
- $\gamma \cdot x$: The formulation where both the residual connection and the bias term β are removed.

6 Case Study

In this study, three human-derived malignant cell lines were utilized: HT-29 (colorectal adenocarcinoma), A2780 (ovarian carcinoma), and KPL-1 (breast cancer). The biological characteristics and origins of these cell lines are summarized as follows:

- **HT-29 (Human Colorectal Adenocarcinoma):** The HT-29 cell line was originally established in 1964 by J. Fogh and colleagues, derived from a primary colorectal adenocarcinoma of a 44-year-old Caucasian female (Fogh and Trempe, 1975). It is widely utilized as a model for intestinal epithelial studies due to its ability to differentiate into mature phenotypes, including mucus-secreting goblet cells. In bioinformatic and functional assays, this cell line provides a robust platform for investigating pattern recognition receptor signaling, cytokine production, and the impact of dietary compounds on gut barrier integrity Martínez-Maqueda et al. (2015).

- **A2780 (Human Ovarian Carcinoma):** The A2780 cell line, derived from an untreated patient with ovarian carcinoma, remains one of the most widely utilized models in gynecological oncology Hamilton (1984). Characterized by an adherent growth morphology and a rapid doubling time, it serves as a critical parental line for investigating the molecular mechanisms of chemoresistance—particularly to platinum-based therapies. While its genomic profile distinguishes it from high-grade serous carcinoma, it remains a gold standard for initial drug efficacy screening and the study of acquired resistance pathways Beaufort et al. (2014).

- **KPL-1 (Human Breast Cancer):** The KPL-1 cell line was established by Kurebayashi et al. from the malignant pleural effusion of a 47-year-old female patient with breast cancer Kurebayashi et al. (1999). KPL-1 is a hormone receptor-positive model, expressing both Estrogen Receptor (ER) and Progesterone Receptor (PR). Compared to the more common MCF-7 cell line, KPL-1 exhibits higher tumorigenicity and invasiveness in nude mice Jäger et al. (1996). Consequently, it serves as a robust model for investigating endocrine therapy resistance and tumor microenvironment interactions.

7 Algorithm

The detailed procedure of the proposed bidirectional environmental condition encoding and modulation is summarized in Algorithm 1.

Algorithm 1 Bidirectional Environmental Condition Encoding and Modulation

Require: Cell line features $\mathbf{c} \in \mathbb{R}^H$, Drug A features $\mathbf{d}_A \in \mathbb{R}^H$, Drug B features $\mathbf{d}_B \in \mathbb{R}^H$
Ensure: Condition-aware modulated features $\mathbf{film}_A, \mathbf{film}_B \in \mathbb{R}^H$

Step 1: Environmental Condition Encoder (ECE)
for drug $i \in \{A, B\}$ with partner drug $j \in \{B, A\}$ **do** ▷ Bidirectional encoding
  $\mathbf{z}_0^{(i)} \leftarrow [\mathbf{c}; \mathbf{d}_j; \mathbf{c} \odot \mathbf{d}_j; \mathbf{c} - \mathbf{d}_j]$ ▷ Multi-perspective interaction representation
  $\mathbf{z}^{(i)} \leftarrow \text{LayerNorm}(\text{GELU}(\mathbf{W}_p \mathbf{z}_0^{(i)} + \mathbf{b}_p))$ ▷ Latent projection
  $\mathbf{z}_{gate}^{(i)} \leftarrow (\mathbf{W}_a \mathbf{z}^{(i)}) \odot \text{GELU}(\mathbf{W}_g \mathbf{z}^{(i)})$ ▷ GEGLU-style gated transformation
  $\mathbf{z}_{bilinear}^{(i)} \leftarrow \sum_{k=1}^{r} \mathbf{w}_k (\mathbf{u}_k^\top \mathbf{c})(\mathbf{p}_k^\top \mathbf{d}_j)$ ▷ Low-rank bilinear interaction
  $\mathbf{z}^{*(i)} \leftarrow \mathbf{z}^{(i)} + \mathbf{z}_{gate}^{(i)} + \mathbf{z}_{bilinear}^{(i)}$ ▷ Residual fusion
  $\mathbf{s}^{(i)} \leftarrow \sigma(\mathbf{W}_2 \, \text{SiLU}(\mathbf{W}_1 \mathbf{z}^{*(i)}))$ ▷ Channel-wise recalibration (SE)
  $\mathbf{con}_i \leftarrow \mathbf{z}^{*(i)} \odot \mathbf{s}^{(i)}$ ▷ Environmental condition vector
end for
Step 2: Residual Feature-wise Linear Modulation
for drug $i \in \{A, B\}$ **do**
  $[\gamma_i, \beta_i] \leftarrow \text{MLP}_{\text{FiLM}}(\mathbf{con}_i)$ ▷ Generate modulation parameters
  $\gamma_i \leftarrow \tanh(\gamma_i)$ ▷ Scaling constraint
  $\mathbf{film}_i \leftarrow \text{LayerNorm}(\mathbf{d}_i + \gamma_i \odot \mathbf{d}_i + \beta_i)$ ▷ Residual FiLM modulation
end for
return $\mathbf{film}_A, \mathbf{film}_B$

References

Corine M Beaufort, Jean CA Helmijr, Anna M Piskorz, Marlous Hoogstraat, Kirsten Ruigrok-Ritstier, Nicolle Besselink, Muhammed Murtaza, Wilfred FJ van IJcken, Anouk AJ Heine, Marcel Smid, et al. Ovarian cancer cell line panel (occp): clinical importance of in vitro morphological subtypes. *PloS one*, 9(9):e103988, 2014.

Jørgen Fogh and Germain Trempe. New human tumor cell lines. In *Human tumor cells in vitro*, pages 115–159. Springer, 1975.

Thomas C Hamilton. Experimental model systems of ovarian cancer: applications to the design and evaluation of new treatment approaches. *Semin Oncol*, 11:285, 1984.

Elke Jäger, Mark Ringhoffer, Hans Peter Dienes, Michael Arand, Julia Karbach, Dirk Jäger, Christiane Ilsemann, Manfred Hagedorn, Franz Oesch, and Alexander Knuth. Granulocyte-macrophage-colony-stimulating factor enhances immune responses to melanoma-associated peptides in vivo. *International journal of cancer*, 67(1):54–62, 1996.

JUNICHI Kurebayashi, T Otsuki, CK Tang, M Kurosumi, S Yamamoto, K Tanaka, M Mochizuki, H Nakamura, and H Soono. Isolation and characterization of a new human breast cancer cell line, kpl-4, expressing the erb b family receptors and interleukin-6. *British journal of cancer*, 79(5):707–717, 1999.

Daniel Martínez-Maqueda, Beatriz Miralles, and Isidra Recio. Ht29 cell line. *The Impact of Food Bioactives on Health: in vitro and ex vivo models*, pages 113–124, 2015.
